# Supplementary material for: A game theoretic analysis of research data sharing
Source: PeerJ. 2015 Sep 8;3:e1242. doi: 10.7717/peerj.1242 (PMC4579014; doi:10.7717/peerj.1242)
Supplement: Appendix S1 [file peerj-03-1242-s001.docx]

**Appendix 1. The calculations to determine *X*, the pool of available datasets, at steady state.**

The rate of change in the pool of available datasets *X* is determined by the rate P_s_ Y_s_ at which datasets P_s_ are added to the pool by the sharing researchers *Y_s_*, and the rate at which the pool-size decays (say to a loss in relevance or a maximum in the storage time). So, we write

$d_{t}X=Y_{s}\cdot P_{s}-q_{x}\cdot X$ (1)

Here the relative decay rate *q_x_* can be associated with the mean life time of the datasets, i.e. the mean life-time of the publications is given as 1/q_x_ . Also, $P_{s}=\frac{1}{T_{s}}$, is the number of papers produced by a researcher, where *T_s_* is the time spent to write it. So, we can write

$d_{t}X=Y_{s}\cdot\frac{1}{T_{s}}-q_{x}\cdot X$ (2)

(where T_s_ is a function of X and Y). If the system is supposed to be at steady state, the change *d_t_X* is zero, i.e. we can write

$0=Y_{s}\cdot\frac{1}{T_{s}}-q_{x}\cdot X$ (3^A^)

i.e.

$q_{x}\cdot X=Y_{s}\cdot\frac{1}{T_{s}}$, (3^B^)

and as the time to produce a paper is given by (SEE EXPRESSION (3) FROM THE MAIN TEXT)

$T_{s}=t_{a}+\frac{t_{d}}{1+f\cdot X}+\left(t_{r}-\frac{t_{r}}{1+f\cdot X}\right)+t_{c}$ (4)

expression (3^B^) becomes

$q_{x}\cdot X=Y_{s}\cdot\frac{1}{\left(t_{a}+t_{c}+\frac{t_{d}}{1+f\cdot X}+t_{r}\frac{f\cdot X}{1+f\cdot X}\right)}$ (5)

In the right hand side of expression (5) we multiply both numerator and denominator with (1+*f·X*)/(1+*f·X*) leading to

$q_{x}\cdot X=\frac{Y_{s}\cdot\left(1+f\cdot X\right)}{\left(\left(t_{a}+t_{c}\right)\left(1+f\cdot X\right)+t_{d}+t_{r}\cdot f\cdot X\right)}$ (6^A^)

which we can write as

$Y_{s}\cdot\left(1+f\cdot X\right)=q_{x}\cdot X\cdot\left(\left(t_{a}+t_{c}\right)\left(1+f\cdot X\right)+t_{d}+t_{r}\cdot f\cdot X\right)$ (6^B^)

Multiplying out all terms, and rearranging the terms results in the second order polynomial in X,

$X^{2}\cdot[q_{x}\cdot f\cdot(t_{a}+t_{c}+t_{r})]+X\cdot[q_{x}\cdot(t_{a}+t_{c}+t_{d})-Y_{s}\cdot f]-Y_{s}=0$ (7)

Since for the above top the constant term is negative, i.e. it equals (– Y_s_ ), it is the upper root which specifies the steady state value X for the available pool of data sets, i.e.

$X=\frac{-B+\sqrt{B^{2}-4AC}}{2A}$ (8)

in which

$A=q_{x}f(t_{a}+t_{c}+t_{r})$ (8^A^)

$B=q_{x}(t_{a}+t_{c}+t_{d})-Y_{s}\cdot f$ (8^B^)

$C=-Y_{s}$ (8^C^)

That is, explicit substitution results in (9)

$$X=\frac{-\left(q_{x}\left(t_{a}+t_{c}+t_{d}\right)-Y_{s}\cdot f\right)+\sqrt{\left(q_{x}\left(t_{a}+t_{c}+t_{d}\right)-Y_{s}\cdot f\right)^{2}-4\left(q_{x}\cdot f\left(t_{a}+t_{c}+t_{r}\right)\right)\cdot\left(-Y_{s}\right)}}{2\left(q_{x}\cdot f\left(t_{a}+t_{c}+t_{r}\right)\right)}$$

(which is expression (5) from the MAIN TEXT)

We finally note that according to (6), for fixed parameters, and fixed size Y_s_, the pool size X_t_ indeed must converge to the steady state value given by (8) or (9).

The script to numerically calculate the values of X up until steady state at fixed parameter values is available in the file 'PoolofavData_v4_app1.R' via http://hdl.handle.net/10411/20328 V4 [Version].
