# Supplementary material for: A game theoretic analysis of research data sharing
Source: PeerJ. 2015 Sep 8;3:e1242. doi: 10.7717/peerj.1242 (PMC4579014; doi:10.7717/peerj.1242)
Supplement: Appendix S2 [file peerj-03-1242-s002.docx]

**Appendix 2.
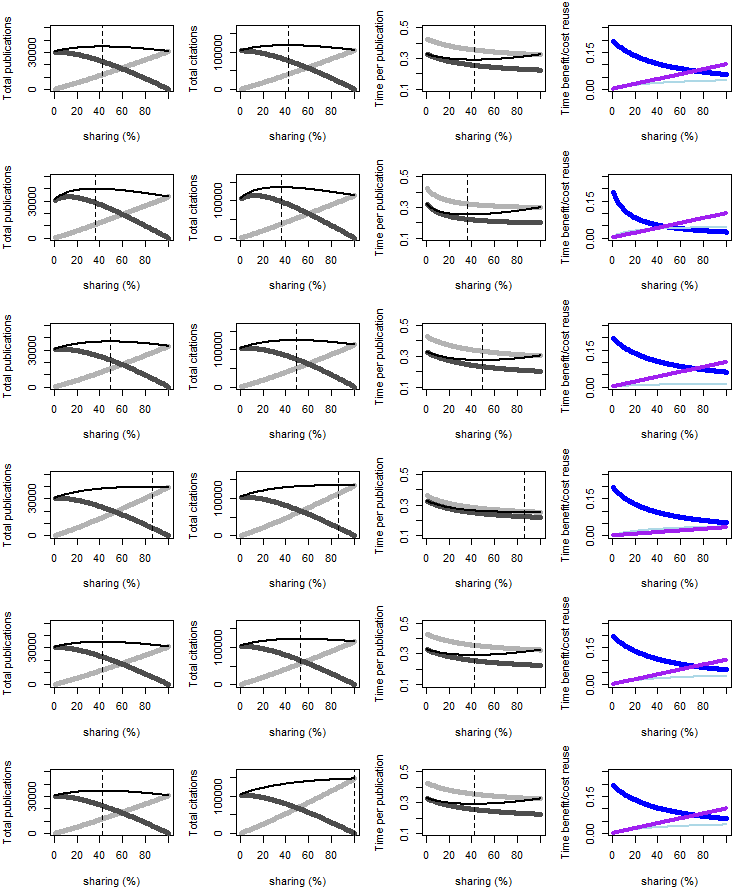
**

Appendix 2. These figures in Appendix 2 are a result of simulations with the same parameters as used for Figure 2 and 3 in the manuscript and with sharing varied in each simulation from 0 to 100% researchers sharing. The figure consists of four results in columns: 1) total publications, 2) total citations, 3) time per publication, 4) the average costs and benefits for a sharing researcher. Thick light-grey lines are the sharing researchers; thick dark-grey lines are not sharing researchers. The thin black line is the average in the community. The black dotted vertical straight line depicts the sharing % at which the average community has maximum value. For the last column, community averages are depicted. The thickest line is the time-cost to produce a dataset, the middle thickest line is the time-cost with sharing, and the thinnest line is the time-cost to process a dataset for reuse.
